# Lack of Maternal Social Capital Increases the Likelihood of Harsh Parenting

**DOI:** 10.3390/children9010099

**Published:** 2022-01-11

**Authors:** Sangwon Kim, Desmond K. Runyan, Yanghee Lee

**Affiliations:** 1 Department of Child Psychology and Education, Sungkyunkwan University, Seoul 03063, Korea; sangwoninkorea@gmail.com; 2 Kempe Center, Department of Pediatrics, University of Colorado, Aurora, CO 80045, USA; Des.Runyan@cuanschutz.edu

**Keywords:** harsh parenting, social capital, children, prevention

## Abstract

Does low maternal social capital increase the likelihood of parents using harsh parenting behaviors? We analyzed random digit dial telephone survey data from 661 female primary caregivers across Colorado. Positive reports of the use of either physically or psychologically harsh parenting methods were classified as harsh parenting. Absence of social capital was assessed within the family and the community; lack of social capital within the family was measured in terms of an absence of support from a partner and an additional caregiver. Absence of social capital within the community was measured as lack of interpersonal resources from neighbors and religious activities. Nearly 30% admitted to one or more physically harsh parenting behaviors in the prior year, and 85.8% reported at least one psychologically harsh parenting behavior. Lower levels of neighborhood connectedness were associated with physically harsh parenting (odds ratio = 1.50). Conflict between partners (odd ratio = 2.50) and the absence of an additional caregiver (odds ratio = 1.88) increased psychologically harsh parenting. One practical implication is that mental health and medical providers should help new parents value, access, or develop social networks within the community to prevent children from experiencing harsh parenting.

## 1. Introduction

In the process of being reared by their parents, children learn how to perceive themselves, others, and society. Parents help children learn to form, understand, maintain, and develop relationships, competencies that they need throughout life [1]. However, children may also be physically or emotionally harmed by parents. Harsh parenting, defined as parents’ verbal or physical acts done to cause children to experience physical or psychological pain and injury for the purpose of correction or punishment [2], is a more socially acceptable way of describing acts that some in society define as abusive.

Hash parenting has been considered acceptable due to traditional views that children are possessions of parents, especially in Asian cultures [3,4]. Although certain positive effects of harsh parenting, such as immediate compliance or reductions in challenging behaviors, have been described [5,6], a number of researchers and child advocates suggest that harsher forms actually constitute child abuse [7,8,9]. As harsh parenting is part of a continuum of child physical or emotional discipline raging from mild to severe in intensity, there is concern that what has been termed harsh punishment is actually child abuse at some point [10]. Empirically, one study which targeted 488 at-risk mothers explained that 67% of mothers did one or more forms of harsh parenting when their child was one year old, and the following year, 87% of mothers reported doing so [8]. There is emerging evidence that harsh parenting rates are declining in many countries and that the most extreme forms of physical punishment are uncommon [11]. However, an estimated 55% of families worldwide still use some form of physical punishment with their children [12,13]. Although 63 countries have outlawed all forms of corporal punishment of children, including in the home [14], harsh punishment or parenting remains common even in these countries [6,15].

Harsh parenting leads to physical, behavioral, emotional, cognitive, and relational difficulties, which seriously impact child wellbeing [6,16,17]. Specifically, children subjected to harsh parenting are more likely to exhibit aggressive behaviors [16,17]. Moreover, harsh parenting has been associated with children’s emotional dysregulation, which may later manifest as maladaptive behaviors [18]. Although many medical and mental health professionals, and the public, tend to pay attention to harsh parenting only when it is tragic or “serious enough” [19], these observations suggest that harsh parenting should be carefully monitored, and that non-harsh parenting should be championed to prevent the predictable harmful consequences of the former.

Parenting is not independent of nor tangential to the surrounding environment; it can be enhanced or undermined by relationships with others [20,21,22]. This aspect is well supported through Bronfenbrenner’s ecological system theory [23], and it is understood that harsh parenting could be shaped or constrained by the contextual surroundings of parents. Recent studies on parenting take familial or neighborhood context into consideration rather than just focusing on parental characteristics, such as socioeconomic status, age, or ethnicity [24]. Family members and neighbors may influence parenting behaviors by functioning as relationship-based resources [15,19,23]. Resources obtained from interpersonal relationships are defined as “social capital” [25].

Social capital is a sociological concept that is difficult to measure, as it results from relationships within the family and in the community. Social capital is usually defined by its functions: generating trust, obligation, and expectations, which then influence people’s behavior [25]. Social capital is embedded in social structures, such as the family or neighborhood; it is usually measured as the degree of cohesiveness in relationships among family members or neighbors [26,27]. Empirical studies have found that when parents experience help, support, or bonding obtained from these cohesive relationships, they are less likely to use harsh or violent methods in disciplining their children [20,21,22,28].

However, the findings of relationships between social capital and parenting are not uniform. While the studies noted above showed that parents who perceived more social capital were less likely to use harsh discipline, other investigators found that social capital had little to do with parenting [29,30]. This discrepancy in studies may be a result of how social capital was measured. Importantly, social capital measured at the individual level could be different from that measured at a group level; an individual might perceive little or no access to social capital even while living in a neighborhood or community known to be prosperous with social capital [31].

Given the dangers of harsh parenting in children’s lives and its use being influenced by the parents’ surrounding environment, it is worthwhile to investigate whether a lack of parental social capital increases harsh parenting. We examined whether a lack of mothers’ social capital obtained within the home and the community is associated with an increased likelihood of physically and/or psychologically harsh parenting. If the relationship between social capital and harsh parenting is strong, mental health and medical professionals can be challenged to help provide environments in their practices and communities where parental social capital can be enhanced in order to prevent harsh punishment and child abuse.

## 2. Materials and Methods

### 2.1. Participants

The Raising Colorado household parenting survey was a computer-assisted telephone-based parenting survey toward mothers across Colorado in the United States. The Carolina Survey Research Laboratory (CSRL) conducted this survey at the University of North Carolina at Chapel Hill. CSRL obtained, from commercial vendors, lists of working telephone numbers of households determined to have children in the home. Numbers to call were selected randomly from these lists. Cell phone numbers were explicitly included. Given that less than two percent of households in Colorado do not have telephone service [32], the coverage rate of this sample design is at least 98%, with the adequately representative of cellphone-only households. Calls were made between December 2013 and May 2014. Out of a total of 21,529 calls, 1576 eligible households (29%) were selected. The criteria included the age of respondents (over 18) and their children (under 18), the state of residence (Colorado), and the primary household language (English or Spanish). All processes, including data collection, were closely monitored by a CSRL supervisor, and incentives were not used in this study because of the potential link of identifying information and sensitive behavior. Among the households, 685 caregivers completed 25 min long interviews upon their informed consent, and interviewers randomly selected an index child if there was more than one eligible child. Professional contact numbers were offered for consultation if they needed any professional help on relevant issues of the survey. A total of 661 responses were utilized for this analysis after excluding cases where a relationship to the index child was other than the child’s parents (e.g., grandparent, egg donor, guardian, or nephew). Missing data were handled using the full information maximum likelihood, which is considered efficient and less biased than other traditional methods (e.g., deletion or mean imputation) [33].

### 2.2. Measures

Harsh parenting was divided into physical and psychological categories. Questions regarding harsh parenting were created based on the expanded version of the World Studies of Abuse in the Family Environment (WorldSAFE) core questionnaire [13], the Parent-Child Conflict Tactics Scale (CTS) [34], and the Dimensions of Discipline Inventory (DDI) [35]. Physically and psychologically harsh parenting measures contained seven questions each (Table 1). There are various perspectives on defining harsh parenting; we explicitly included “spanking” as a form of physically harsh parenting in our analyses. Examples of physically harsh parenting included ‘hit child’s buttocks with an object’ and ‘slapped on the face or the back of the head’. Cronbach’s alpha of physically harsh parenting was 0.504. Examples of psychologically harsh parenting included ‘threatened to leave or abandon the index child’ and ‘cursed or swore at the index child’. Cronbach’s alpha of psychologically harsh parenting was 0.616. The response of two types of harsh parenting was coded as ‘1′ if the parent used one of the approaches listed in the question more than ‘once or twice in the past year,’ and ‘0′ represented otherwise.

The absence of social capital was assessed within the family and the community; a lack of social capital within the family was measured in terms of an absence of support from a partner and an additional caregiver [26,27,30]. First, each respondent was asked to report, using six items, whether she or her partner had insulted, threatened, or slapped each other in the past year. The response options were ranged from ‘never happened (coded as 0)’ to happened ‘more than 20 times in the past year (coded as 6)’, and reports were totaled across partners. The internal consistency of the six items was 0.573. In addition, social capital accrued when an additional caregiver who could nurture the child was in the home; we calculated a ratio of the number of all children to responsible adults in the household. If the ratio was lower than 1, it represented less social capital (coded as ‘1′), and if the ratio was above 1, it denoted having social capital.

Lack of interpersonal resources from neighbors and religious service participation was used to measure the absence of community-based social capital [26,27,30]. Neighborhood connectedness was measured using five questions, each coded on a 4-point scale ranging from ‘very much’ to ‘not at all (e.g., would you say that your neighbors watch out for each other’s children?)’. The internal consistency of the five items was 0.821. Religious service participation, another marker of social capital, was measured based on the frequency of monthly participation. Referring to a prior study [28], participating in religious services < 8 times a month was coded as ‘1′ for less social capital, whereas ‘0′ represented having social capital.

Individual, familial, and neighborhood characteristics were introduced as control variables [5,22]. Individual control variables included the ages of the index child and the mother; maternal mental and behavioral health diagnoses (respondents with no diagnosis constituted the reference group); maternal education; maternal race/ethnicity (White/Caucasian as the reference group); and the child’s gender (girl as the reference group). Familial control variables included whether or not the household received public assistance (Medicaid, Temporary Assistance to Needy Families, or Women, Infants & Children program, respondents without public assistance as the reference group). Neighborhood control variables included residence area (living in a rural area as the reference group).

### 2.3. Analytic Process

We used multivariate logistic regression to explore the relationship between a lack of maternal social capital and the likelihood of harsh parenting using Mplus 8.3 [36]. This study weighted the sample by estimates of socioeconomic status and race/ethnicity to represent the state of Colorado in the USA.

## 3. Results

Of the 661 female primary caregivers, approximately 83% were White/Caucasian, with a mean age of 43.02 years and a mean education of 15 years. About 14.1% of the families received public assistance. Almost half of the index children were female, with a mean age of 10.70 years. Nearly 30% of the parents admitted to using at least one physically harsh parenting behavior, and 85.8% reported using at least one psychologically harsh parenting behavior in the last year. A description of the respondents and harsh parenting is presented in Table 1.

Table 2 presents the number and percentages of affirmative responses for individual items of harsh parenting. Mothers used psychologically harsh parenting behaviors more often than physically harsh parenting behaviors. Among physical behaviors, mothers used spanking most frequently (25.1%), with fewer than 6% of them using other physical methods in the prior year. In contrast, mothers reported more frequently engaging in shouting (84.3%), cursing (32.8%), or making their children feel ashamed (31.2%). Multivariate logistic regression was used to explore whether a lack of maternal social capital influenced the likelihood of committing either physically or psychologically harsh parenting.

Detailed findings controlling for individual, familial, and neighborhood variables are presented in Table 3. Concerning physically harsh parenting, although respondents who have conflict between partners or no additional caregiver who could raise children tended to parent their children in a physically harsher mode, no statistical significance was found. With regard to social capital outside the family, respondents who reported less neighborhood connectedness were more likely to report committing a physically harsh parenting method toward their children (odds ratio = 1.50). Participating in religious services did not significantly affect the likelihood of committing psychologically harsh parenting.

Psychologically harsh parenting was only influenced by social capital within the family, not social capital outside the family. The more conflict between partners (which indicates a lack of mother’s social capital from the partner), the more likely they are to use psychologically harsh parenting (odds ratio = 2.50). Other social capital accrued within the family also mattered; mothers with less access to additional help in caring for their children in the household were more likely to be involved in psychologically harsh parenting than those who had additional caregivers (odds ratio = 1.88).

## 4. Discussion

The overall parenting pattern observed in this Colorado sample was similar to patterns in an earlier international study [13]. Most of the respondents reported that they did not use physically harsh parenting methods other than ‘spanking’. However, most parents reported using psychologically harsh parenting, including ‘shouting, yelling, or screaming’. Many pediatric practices and communities have developed parental education programs to help parents with parenting education and prevent child abuse. Current and new parenting education efforts should raise awareness about the potential for commonly used harsh discipline practices (i.e., ‘spanking’, ‘shouting, yelling, or screaming at children’, and ‘cursed or swore at child’) escalating to child abuse [5,7]. As parents with limited parenting repertoires may use more violent parenting methods [37], education regarding alternative parenting methods (e.g., positive parenting) should be combined with awareness-raising about the dangers of harsh parenting [38].

Having a lower sense of connectedness to neighbors appears to be associated with a higher likelihood of physically harsh parenting behaviors. Connectedness with neighbors may give mothers more sense that they are either supported or monitored; both may lead to less harsh parenting behavior. Coohey (2000) contends that the community relationship may play a supervisory role whereby it compels mothers to refrain from using physically harsh parenting methods on their children. In the context of building stronger community relationships, establishing social programs, or even a site, within a pediatric practice where interaction among neighbors is encouraged to enhance social capital may benefit children. Group well-child primary care is one example [39]. In this context, the process of forming social relationships may vary depending on the community or the residential area in which they live [40], consideration should be given to this.

Psychologically harsh parenting was only influenced by social capital within the family. Previous studies have found that conflicts in marriage may hinder effective parenting practices [29,41]. In this context, programs or supportive services directed at strengthening partner relationships could be considered, along with education about the harms from harsh parenting. This is because the weakening of maternal social capital due to repeated conflicts between partners can eventually lead to harsh discipline toward children, which further diminishes the children’s social capital obtained from parents within the family. Efforts toward the further engagement of fathers in parenting classes and well-child care should also be considered by medical or social service providers [42]. Of note, our findings may justify screening for intimate partner violence in pediatric practice even though evidence for reductions in violence against women has not been shown to result from domestic violence screening for women [43].

Mothers with no additional caregivers were more likely to engage in psychologically harsh parenting. This finding could be inferred from previous studies in that having access to additional support in childrearing can lead to enhanced emotional stability, which can result in less violent parenting [26,44,45]. To maximize the benefit of additional caregivers, training to harmonize parenting methods among caregivers could be utilized. This is because new conflicts may arise if there are simply enough caregivers per child without an agreement among caregivers. In cases where additional caregivers are not practical, community child care centers or parenting support groups may provide similar support. Additionally, monitoring the home safety level could be considered through existing programs within the region, such as SafeCare^®^ Colorado [44].

Notably, our findings differ from prior research, which found that religious service participation helps reduce harsh parenting [24,45]. These unexpected findings may be a limitation stemming from our measurement of religious activities’ frequency per month. The underlying relationship may not always be linear; more occasions attending religious services may not equal more social engagement. Further use of religious service participation as a social capital measure may require considering other religious participation aspects.

This study was meaningful in exploring the relationship between the lack of a caregiver’s social capital and the use of harsh disciplinary practices. However, a cautious interpretation of the results is warranted. First, probability sampling was used, and the response rate was not very high. This feature leaves the possibility that non-respondents could be at a higher or lower risk for using harsh parenting methods on their children. Besides, most of our respondents were characterized as highly educated Whites/Caucasians with high incomes, which is considered a less risky group. It also suggests the possibility that this research model may not be applied in different cultures. In future studies, using a variety of approaches to recruit participants from various backgrounds, including online surveys, could compensate for this limitation. Second, in terms of measurements, harsh parenting was measured using a frequency basis in retrospective mode. This approach has a notable weakness concerning memory reliability and social desirability tendency. One possible countermeasure for this limitation could be merging a self-report with either multiple informants (e.g., children or partner) or formal administrative sources. In particular, if the partner’s responses were considered after the mother’s safety was secured, the reliability of the mother’s self-report would be supplemented. Also, richer results could have been drawn in that the partner is one of the members of the family system as well as another subject of child-rearing. Third, this study included all cases of children under 18 to examine the relationship between mothers’ social capital and the likelihood of harsh discipline methods, controlling for their background characteristics, including children’s age. However, how harshly their parents discipline their children can be distinguished according to children’s age [46]. In future studies, building on this study, it will be helpful if grouping children by age can devise a more targeted intervention strategy. Fourth, there is a high probability that both physically and psychologically harsh parenting co-occur. This might contribute to a differential influence each form of social capital has on each harsh parenting form presented in this study. Thus, in future studies, the relationship between social capital and harsh parenting should be revisited while considering the co-occurrence of physically and psychologically harsh parenting. Fifth, the data collected between 2013 and 2014 could be another limitation. However, the finding of harsh parenting patterns did not differ from the previous study [13]; therefore, it can be inferred that harsh parenting methods do not change significantly.

Our findings suggest that parents should access an environment where social capital is well-established to prevent children from experiencing harsh parenting. Assisting mothers in accumulating social capital may be a useful approach to reduce harsh discipline towards children. Medical or social service providers can assist with this through their practices.

## Figures and Tables

**Table 1:** Descriptive summary of model variables (N = 661).

Variable	Mean	Standard Deviation	Range	
			Min	Max
Harsh parenting				
Physically harsh parenting	0.30	0.55	0	1
Psychologically harsh parenting	0.86	0.35	0	1
Lack of social capital within family				
Violence between intimate partner	0.44	0.56	0	2.80
Additional caregiver ratio < 1	0.64	0.48	0	1
Lack of social capital outside family				
Less neighborhood connectedness				
Neighbors concerned your wellbeing	1.84	0.94	1	4
Neighbors watch out each other’s children	1.65	0.81	1	4
You can count on neighbors	1.48	0.77	1	4
Neighbors are willing to help others	1.52	0.60	1	4
Neighbors can be trusted	1.59	0.63	1	4
Religious service participation < 8 times a month	0.93	0.26	0	1
Control variables				
Individual controls				
Maternal age	43.02	7.89	18	62
Maternal mental & behavioral diagnoses ^a^	0.06	0.23	0	1
Maternal race/ethnicity (non-White/Caucasian) ^b^	0.17	0.38	0	1
Maternal education	15.39	2.38	6	20
Child’s gender (male) ^c^	0.50	0.50	0	1
Child’s age	10.70	4.83	0	17
Familial controls				
Public assistance ^d^	0.14	0.35	0	1
Neighborhood controls				
Residence (rural area) ^e^	0.12	0.32	0	1

^a^ Reference group consists of respondents with no diagnosis ^b^ Reference group is White/Caucasian. ^c^ Reference group is female. ^d^ Reference group was the family that receives public assistance (Medicaid, Temporary Assistance to Needy Families, or Women, Infants & Children program). ^e^ This information was originally collected by asking what county they live in and was re-grouped into rural and urban areas based on the Colorado County Designation. Nineteen respondents reported residing in one of the 12 exceptionally rural counties referred to as frontier counties in Colorado. These respondents were re-categorized as rural as there were no statistical differences between rural and frontier residents in harsh parenting. The reference group consisted of respondents residing in an urban county.

**Table 2:** Measures of harsh parenting in the last year, N (%).

Harsh Parenting	Occurred	Not Occurred
Physically harsh parenting	197 (29.8)	464 (70.2)
Hit buttocks with objects	18 (2.7)	643 (97.3)
Hit somewhere else with objects	13 (2.0)	648 (98.0)
Kicked	3 (0.5)	658 (99.5)
Spanked on the buttocks with hand only	166 (25.1)	494 (74.7)
Pinched	21 (3.2)	639 (96.7)
Slapped on the face or the back of the head	37 (5.6)	624 (94.4)
Beat (hit over and over again with an object)	-	661 (100.0)
Psychological harsh parenting	567 (85.8)	94 (14.2)
Threatened to leave or abandon	27 (4.1)	620 (93.8)
Shouted, yelled, or screamed at child	557 (84.3)	104 (15.7)
Cursed or swore at child	217 (32.8)	443 (67.0)
Threatened to kick out of the house or send away	23 (3.5)	623 (94.3)
Called child names like stupid, ugly, or useless	25 (3.8)	636 (96.2)
Refused to speak as a punishment	117 (17.7)	543 (82.1)
Made child feel ashamed	206 (31.2)	450 (68.1)

**Table 3:** Lack of social capital, physically, and psychologically harsh parenting ^a^.

Variable	Physically Harsh Parenting			Psychologically Harsh Parenting		
	Odds Ratio	B (SE)	C.I.	Odds Ratio	B (SE)	C.I.
Lack of social capital within family						
Violence between intimate partner	1.10	0.10 (0.21)	−0.31–0.51	2.50	0.91 ** (0.32)	0.28–1.55
Additional caregivers’ ratio < 1	1.36	0.30 (0.25)	−0.18–0.78	1.88	0.63 * (0.26)	0.11–1.15
Lack of social capital outside family						
Less neighborhood connectedness	1.50	0.40 * (0.20)	0.01–0.80	1.13	0.12 (0.26)	−0.38–0.62
Religious service participation (<8 times a month)	1.20	0.18 (0.44)	−0.68–1.04	1.27	0.24 (0.44)	−0.62–1.10
Individual controls						
Maternal age	0.99	−0.02 (0.05)	−0.06–0.03	1.01	0.01 (0.02)	−0.04–0.06
Maternal mental & behavioral diagnoses ^b^	0.96	−0.04 (0.56)	−1.13–1.05	1.51	0.41 (0.64)	−0.83–1.66
Maternal race/ethnicity (non- White/Caucasian) ^c^	1.62	0.49 (0.27)	−0.04–1.01	1.14	0.13 (0.33)	−0.53–0.78
Maternal education	0.98	−0.02 (0.05)	−0.06–0.03	1.04	0.04 (0.06)	−0.09–0.16
Child’s gender (male) ^d^	1.37	0.31 (0.22)	−0.11–0.74	1.27	0.24 (0.26)	−0.27–0.75
Child’s age	0.85	−0.17 *** (0.03)	−0.23–−0.10	1.04	0.04 (0.04)	−0.04–0.12
Familial controls ^e^						
Public assistance ^f^	0.56	−0.56 (0.35)	−1.26–0.10	0.94	−0.07 (0.38)	−0.82–0.69
Neighborhood controls						
Residence (rural area) ^g^	1.78	0.57 (0.34)	−0.04–1.01	1.24	0.22 (0.39)	−0.53–0.78

Standard errors in parentheses. * *p* < 0.05. ** *p* < 0.01. *** *p* < 0.001. ^a^ Weighted logistic regression was conducted based on Colorado population estimates for socioeconomic status and race/ethnicity. ^b^ Reference group is respondents with no diagnosis. ^c^ Reference group is White/Caucasian. ^d^ Reference group is female. ^e^ Having a partner was designed as a familial covariate but was deleted as the variance was zero. ^f^ Reference group includes respondents with public assistances (Medicaid, Temporary Assistance to Needy Families, or Women, Infants & Children program). ^g^ Reference group is respondents residing in urban areas.
